# Disturbed oscillatory brain dynamics in subcortical ischemic vascular dementia

**DOI:** 10.1186/1471-2202-13-85

**Published:** 2012-07-24

**Authors:** Elisabeth CW van Straaten, Willem de Haan, Hanneke de Waal, Philip Scheltens, Wiesje M van der Flier, Frederik Barkhof, Ted Koene, Cornelis J Stam

**Affiliations:** 1 Department of Clinical Neurophysiology, VU University Medical Center, de Boelelaan 1118, Amsterdam, 1081 HV, Netherlands; 2 Neurology and Alzheimer Center, VU University Medical Center, Amsterdam, Netherlands; 3 Epidemiology & biostatistics, VU University Medical Center, Amsterdam, Netherlands; 4 Radiology, VU University Medical Center, Amsterdam, Netherlands; 5 Medical Psychology, VU University Medical Center, Amsterdam, Netherlands

**Keywords:** EEG, Oscillations, Spectral analysis, Relative power, Vascular dementia, Cognition, White matter

## Abstract

**Background:**

White matter hyperintensities (WMH) can lead to dementia but the underlying physiological mechanisms are unclear. We compared relative oscillatory power from electroencephalographic studies (EEGs) of 17 patients with subcortical ischemic vascular dementia, based on extensive white matter hyperintensities (SIVD-WMH) with 17 controls to investigate physiological changes underlying this diagnosis.

**Results:**

Differences between the groups were large, with a decrease of relative power of fast activity in patients (alpha power 0.25 ± 0.12 versus 0.38 ± 0.13, p = 0.01; beta power 0.08 ± 0.04 versus 0.19 ± 0.07; p<0.001) and an increase in relative powers of slow activity in patients (theta power 0.32 ± 0.11 versus 0.14 ± 0.09; p<0.001 and delta power 0.31 ± 0.14 versus 0.23 ± 0.09; p<0.05). Lower relative beta power was related to worse cognitive performance in a linear regression analysis (standardized beta = 0.67, p<0.01).

**Conclusions:**

This pattern of disturbance in oscillatory brain activity indicate loss of connections between neurons, providing a first step in the understanding of cognitive dysfunction in SIVD-WMH.

## Background

White matter hyperintensities (WMH), especially when severe, can be associated with cognitive impairment and dementia (as a subgroup of subcortical ischemic vascular disease and dementia SIVD) [1]. However, since they are also very frequently seen on cerebral magnetic resonance imaging (MRI) of subjects without cognitive impairment, one of the challenges is to understand when and how these lesions lead to dementia. Analysis of brain activity may prove to be a useful additive tool in addition to neuropsychological and radiological examinations. Neuronal oscillatory activity, as recorded with electroencephalography (EEG), reflects local synaptic activity [2]. Changes in relative power of this activity (mainly slowing of the dominant frequency) are seen in several types of dementia, such as Alzheimer’s disease (AD) [3], and dementia with Lewy bodies and Parkinson’s dementia [4]. In most EEG studies on vascular dementia (VaD), subjects with subcortical dementia as well as dementia after cortical stroke were analysed together [5-8]. In these studies, slowing of posterior dominant activity was the main finding. Only few studies concentrated on SIVD only, but these included WMH as well as lacunar infarcts. Gawel et al. [9] reported a reduction in alpha power and increase in theta and delta power as expressed as lower alpha/slow wave ratio, and this ratio also had a correlation with mini mental state examination (MMSE). Another study found a lower peak frequency with spectral analysis in subjects with subcortical VaD compared to controls [10]. The slowing was comparable or less pronounced than that observed in Alzheimer’s disease. The proportion of subjects with WMH only was not mentioned in these studies, and therefore the relative contribution of WMH cannot be appreciated. As far as we are aware, no study has focused on (quantitative) EEG analysis in SIVD-WMH only.

Hypothetically, WMH can disturb the functional connections by impairment of the anatomical connections (the white matter tracts), globally as well as regionally. We therefore set out to find differences in power spectra as a measure of local synaptic processing and connectivity in subjects with SIVD-WMH, compared to cognitively intact individuals.

## Results

### Demographic data and mean relative power

Table 1 shows a summary of the subject characteristics. No major differences were found between the groups with respect to relevant medication use: benzodiazepines: two patients, three controls; antipsychiatric drugs: one patient; antiepileptic drugs: one patient.

**Table 1:** Baseline demographic and clinical characteristics of SIVD-WMH patients and controls

	**SIVD-WMH patients (n = 17)**	**Control subjects (n = 17)**
Age (yrs )	74.6 ± 8	74.4 ± 8
Sex ratio (m/f)	9/8	9/8
WMH score (Fazekas)	3 ± 0	1 ± 1
MMSE score	22 ± 5	28.5 ± 1
RAVLT immediate recall score	10 ± 12	32 ± 14
RAVLT delayed recall score	1 ± 2	6 ± 4
Trailmaking A (sec)	168 ± 116	45 ± 13
Trailmaking B (sec)	653 ± 320	105 ± 42
Verbal fluency (number / 60 sec)	20 ± 5.7	10 ± 4.8

Values are expressed as mean ± SD unless otherwise indicated.

**Figure 1:**
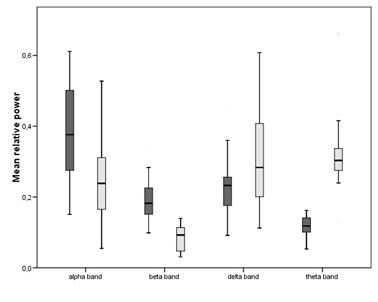
**Box plots of mean relative power (SD) for controls and patients in the different frequency bands.** Light bars: patients, dark bars: controls.

Differences in relative powers between groups were large and statistically significant in all frequency bands (Figure 1). In patients, a decrease in faster activity was found (alpha power 0.25 ± 0.12 SD versus 0.38 ± 0.13, p = 0.01; beta power 0.08 ± 0.04 SD versus 0.19 ± 0.07, p<0.001) and an increase in relative power of slow activity (theta power 0.32 ± 0.11 SD versus 0.14 ± 0.09, p<0.001; delta power 0.31 ± 0.14 SD versus 0.23 ± 0.09, p = 0.04). Four subjects in the control group had a WMH Fazekas score >1. When those were left out of the analysis, the control group had a higher alpha power and lower theta and delta power (change in all cases with 0.01). The p-values were mostly unchanged, however in the delta band p-value went up to 0.06.

**Figure 2:**
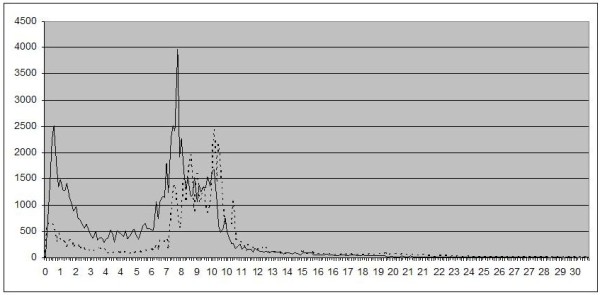
**Mean power spectra of EEG activity at the location O2 (right occipital).** X-axis: frequency in Hz, Y-axis: absolute power. Solid line: patients, dotted line: controls.

Figure 2 shows the quantitative analysis of EEG activity as power spectra. It can be seen that in the patients, the peak is at a lower frequency, and also the largest proportion of the total activity is in the lower frequencies. Other locations (O1, Fz, Cz, and Pz) were evaluated and equal in the distribution of activity.

### Regional relative power

**Figure 3:**
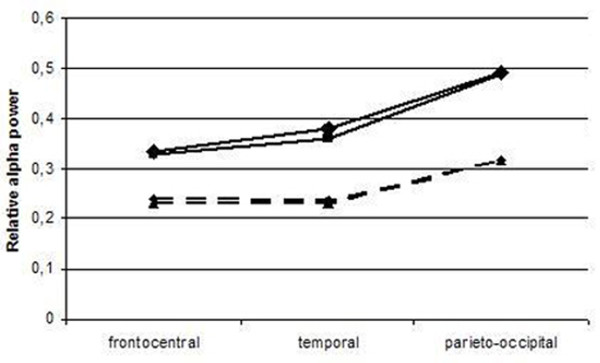
**Mean relative alpha power by brain region.** Dotted lines: patients, solid lines: controls. ▲: right hemisphere ♦: left hemisphere

The results of ANOVA are shown in Table 2. For all frequency bands, main effects of group and brain region were found on relative power. In addition, an interaction between group and brain region was found in the alpha band, indicating that the differences between the groups differed significantly for different brain regions. Figure 3 shows the regional differences between groups in the alpha band. The largest difference between the patients and the controls was in the posterior brain regions, demonstrating that the decrease in alpha power in patients is most pronounced in these areas. In the beta band, the interaction effect nearly reached statistical significance, with highest loss of relative power in the frontal and parieto-occipital regions.

**Table 2:** Analysis of variance (repeated measures) for regional mean relative power values in each frequency band.

	**Within subjects**		**Between subjects**
	**Area**	**Area x Group**	**Group**
Alpha band	F [2.968] = 49.89^*^	F[2.968] = 4.66 ^*^	F[1] = 8.43 ^*^
Beta band	F[3,738] = 12.67 ^*^	F[3,738] = 2.41	F[1] = 37.05 ^*^
Delta band	F[3,772] = 21.36 ^*^	F[3,772] = 1.89	F[1] = 4.65 ^*^
Theta band	F[3,645] = 3.66 ^*^	F[3,645] = 0.87	F[1] = 27.67 ^*^

^*^ p<0.05.

Degrees of freedom are printed between square brackets.

### Relationship to cognitive measures

As can be expected, patients performed worse on the cognitive tests. To relate EEG activity to cognition, regression analysis was performed in this group. A significant relationship was found between MMSE and RAVLT delayed recall, and beta power: lower relative beta power was predictive of lower MMSE (standardized beta = 0.67, p = 0.01) and lower score on the Rey auditory verbal learning test (RAVLT) delayed recall (standardized beta = 0.65, p = 0.01). Interestingly, we found no relationship between EEG measures and the executive measures (Trailmaking tests A and B and fluency). Also, no effect was found of power of other frequency bands on MMSE.

### Discriminative power

Overall, discriminative power was high for all frequency bands, reflecting the large differences between the groups. Highest percentage of correctly classified subjects was found in the beta band. A cut-off value of 0.14 resulted in a sensitivity of 0.82 and a specificity of 1.0 (area-under-the-curve 0.95). For the relative theta power optimal cut-off was 0.20 with an area-under-the-curve of 0.89, sensitivity of 0.94 and specificity of 0.88. For the relative alpha and delta powers most favourable cut-off values were 0.24 and 0.27 respectively with an area-under-the-curve of 0.76 and 0.65, sensitivity of 0.94 and 0.53, and specificity of 0.53 and 0.82 respectively.

## Discussion

We found large differences in relative power between SIVD-WMH patients and controls. EEGs of patients had a larger percentage of slow (delta and theta) oscillatory activity and a clear reduction of the faster (alpha and beta) power. The findings were significant for the global relative powers as well as for several brain regions separately. However, differences were not equal for each brain region: differences in relative alpha power were significantly larger in the parieto-occipital regions. The extent of the differences between the groups was also illustrated by the large area-under-the-curve of the ROC curves (especially in the beta and theta band). Furthermore, a positive relationship was found between relative beta power and MMSE. Relative powers had otherwise no significant relationship with the other cognitive measures that are usually found affected in SIVD (Trailmaking test A and B and word fluency). The findings are in line with previous research [9,11].

The strength of the current study is the relatively pure group of subjects with SIVD-WMH. Previous EEG studies on small vessel disease included subjects with lacunar infarcts as well as WMH. Since these lesions have a different clinical and radiological appearance, it can be hypothesized that they, or their underlying cause, may exert a different effect on electrical activity of the brain as well, and may have a different relationship to cognitive deficits. In this study, the conservative National Institute of Neurological Disorders and Stroke and Association Internationale pour la Recherché et l’Enseignement en Neurosciences (NINDS-AIREN) criteria were applied for diagnosing VaD, securing a high specificity [12]. Moreover, all subjects were thoroughly examined and the results multidisciplinary evaluated to exclude other or concomitant causes of dementia, especially AD, as much as possible. The design of this study was cross-sectional and retrospective. This has some well-known drawbacks, and remarks on causation of EEG slowing by WMH are thus speculative. The control group cannot be regarded as completely healthy because subjective memory complaints are a risk factor for the development of dementia in the future. The choice for these controls might have lead to underestimation of the results increasing the risk of type II error. However, significant differences were found between the groups, so that we feel that the analysis was powerful enough. Additionally, we felt that this group of subjects is clinically relevant since this is the group that needs to be distinguished from individuals with dementia in a memory clinic setting. Furthermore, the controls were not totally free of WMH. These lesions were found to be very common in the elderly with 8% of a general elderly population having no WMH at all (de Leeuw et al., 2001). We therefore regarded the lesion load (Fazekas score 0–2) of the controls not out of proportion. In a sub-analysis, we compared the patients to controls with none or only mild WMH (Fazekas score 0–1) and we found that the differences between the groups were larger, with the control group having a larger proportion of faster activity. This gives some strength to the hypothesis that WMH has an effect on brain activity, although numbers are too small for definitive conclusions.

Relative power of EEG oscillatory activity is likely reflecting the level of coupling between local groups of excitatory and inhibitory neurons. The exact characteristics and direction of interactions cannot be assessed. However, oscillations in the highest frequency bands (beta and gamma activity) have been reported to be mainly mediated by reciprocal cortico-cortical connections, especially, but not exclusively, of neurons in the same cortical area and are associated with several cognitive processes (among which attention, interpretation of perception, and language) [13]. Lower frequency oscillations appear to be dominantly generated by subcortical structures (especially the thalamus) and interactions between cortical and subcortical structures. They tend to occur at longer distances than the fast oscillations [14]. We found a decrease in relative beta power and an increase of the slower frequencies in the patients globally as well as in the regional calculations. This implies that at least shorter distance synchrony of faster oscillations is diminished. To what extent the long distance functional connections are compromised in SIVD-WMH remains to be studied.

We found no relationship between relative power and executive function and speed (indicative of cognitive performance in VaD). Lack of sensitivity of the tests can play a role. On the other hand, our results suggest that these cognitive processes might be mediated differently, with less effect on local connectivity. Other processes than local dysfunction only seem to play a role in executive function. In brain tumors, a global effect on executive function was found, irrespective of the site of the lesion [15]. We speculate that this might also be the case in WMH to some extent.

Regional analyses revealed statistically significant differences between groups for all brain regions, with a very symmetrical distribution between the hemispheres. An interaction was found between group and brain region for the alpha band. The difference in relative alpha power between patient and control group was more outspoken in the posterior and temporal regions. The posterior alpha oscillations are a reflection of the posterior dominant rhythm of the visual system, usually referred to as a ‘stand-by’ state rather than activation. The EEG findings are not specific for SIVD-WMH. Slowing of the dominant rhythm is found in many other neurological conditions, including other types of dementia (Alzheimer’s disease, dementia with Lewy bodies) and encephalopathy (metabolic, or hypoxic encephalopathy) [16,17]. The disappearance of faster oscillations and increase of slow activity reflects some universal mechanism that in modelling studies could be replicated by reducing coupling strength between neuronal units. Weakening of the negative feedback loop of inhibitory and excitatory neurons could underly this general phenomenon [18-20]. It might be hypothesized that in our group of subjects with SIVD-WMH, the vascular lesions are involved in the causal mechanisms of loss of connectivity, explaining the slowing of the EEG activity, although this remains to be studied. Of interest is that SIVD-WMH, with lesions located in the regions of white matter tracts (connecting cortical and subcortical gray matter on a relatively large distant), seemingly have a measurable effect on EEG oscillatory activity as a reflection of local cortical function. This finding leads on one hand to the question how this effect is established. On the other hand might this give directions to a possible mechanism underlying the cognitive deficits in this type of dementia. These results suggest that future studies, also on long distance connectivity, are therefore needed.

## Conclusion

WMH-based vascular dementia patients show a marked slowing of oscillatory activity, as measured with EEG. This type of dementia, that is characterized by widespread subcortical lesions, is therefore associated with changes in local cortical neuronal function. Relationships with neuropsychological function is known to be only weak. However, decrease of faster oscillations was associated to worse cognitive performance in this group of subjects. This may be a step in the understanding how subcortical lesions lead to cortical dysfunction in SIVD-WMH.

## Methods

### Participants

We included 17 SIVD-WMH patients and 17 controls. All subjects were referred to the Alzheimer Center of the VU University Medical Center between 2003 and January 2010 and had undergone diagnostic workup according to the local protocol, including physical, neurological, and laboratory examinations, neuropsychological testing, brain MRI (or, if not possible, computed tomography (CT)) and resting-state EEG. All subjects or their formal representatives gave written informed consent for the storage of the results of the examinations in a local database and for the use of the data for research purposes. This protocol was in agreement with the WMA declaration of Helsinki, and approved by the ethical review board of the VU University Medical Centre. During a multidisciplinary consensus meeting, the SIVD-WMH patients were diagnosed in two steps. First, the diagnosis VaD was made using the NINDS-AIREN criteria [21]. Signs include a dementia syndrome with memory impairment and at least two other cognitive domains affected, and signs of cerebral vascular disease. Second, the vascular brain lesions on imaging consisted of extensive and confluent WMH. This was locally operationalized as a minimum age-related white matter changes (ARWMC) score three in at least two of the four frontal and parietal regions, and at least a score two in the remainder of the four regions [22,23]. Other causes for VaD (cortical infarctions in strategic areas, multiple lacunes) were excluded, as were other MRI abnormalities possibly interfering with the diagnosis. Additionally, the subjects did not meet the criteria for AD [24]. As a result of the described selection, all subjects had a severe global WMH score (grade 3 according to the Fazekas rating scale, in the deep white matter as well as periventricularly) [25] and no other suspected cause for the dementia syndrome. The controls were individuals from the same database who underwent the screening because of subjective cognitive complaints but who had no cognitive abnormalities on testing. They were individually matched for age and gender to the patients. Medication that could influence EEG findings were scored in the following categories: benzodiazepines, antipsychiatric drugs, and antiepileptic drugs.

### EEG and signal analysis

At the first visit resting state EEG of 20 minutes was recorded in all subjects with OSG digital equipment (Brainlab®; OSG b.v. Belgium). Twenty-one electrodes were placed according to the 10–20 international system (Fp2, Fp1, F8, F7, F4, F3, T4, T3, T2, T1, C4, C3, T6, T5, P4, P3, O2, O1, Fz, Pz, and Cz). Electrode impedance was below 5 kΩ when started. Filter settings were time constant 1 s, and low pass filter 70 Hz. Sample frequency was 500 Hz and analog–digital precision was 16 bit. Subjects were seated in a slightly reclined chair in a sound-attenuated, normally lit room and were kept awake as much as possible during the recording. For each subject, four epochs (average reference montage) of almost 10 seconds each were selected on the basis of signal quality (artifact-free) and patient status (eyes closed, awake), then converted to ASCII format and read into BrainWave analysis software (version 0.8.51 for the relative power calculations and version 0.9.70 for the power spectrum calculations), Stam 2010; available at: http:/home.kpn.nl/stam7883/brainwave.html). For each epoch we calculated relative power of each frequency band (delta 0.5–4 Hz, theta 4–8 Hz, alpha 8–13 Hz, and beta 13–30 Hz) with Fast Fourier Transformation (FFT). This was done for the mean of all channels (global power) as well as for several brain regions separately (frontocentral right (Fp2, F4, C4) and left (Fp1, F3, C3), temporal right (F8, T4, T6) and left (F7, T3, T5) and parieto-occipital right (P4, O2) and left (P3, O1)). Mean relative power per patient was calculated by taking the mean of the four epochs. Gamma band (> 30 Hz) activity was left out of the analyses because we felt this could not reliably be distinguished from muscle artifact [26].

### Neuropsychological tests

To asses the relationship of EEG with cognitive functioning, we used the MMSE [27] as a global cognitive measure, and Trailmaking A and B [28] and verbal fluency (animals) [29] tests for executive functioning. The RAVLT (sum of five learning trials for immediate recall and a delayed recall trial after 20 minutes) was used for the assessment of memory function [30].

### Statistical analysis

All analyses were performed at a significance level of 0.05 (two-tailed). Analysis was done using the SPSS 14.0 software package (SPSS Inc., Chicago, IL). Normal distribution of the relative power values was tested with Kolmogorov-Smirnov test, small deviations from normality were accepted. Mean relative powers were compared between subject groups using the Student’s t-test for paired data. Secondly, subjects in the control group with a Fazekas score > 1 were disregarded and the analysis rerun (with the Student’s t-test for independent samples) to correct for the effect of larger lesion load in the control group. We used univariate analysis of variance (ANOVA) for repeated measures for the interaction of subject group and brain regions (brain region as the within subject factor and group as the between subjects factor), with the Greenhouse-Geisser correction for sphericity. Again, this was done for the whole group as well as the group without Fazekas score 2 controls. Linear regression analysis was performed with relative powers as independent variables, age and gender as co-variates, and cognitive measures (MMSE, RAVLT, Trailmaking tests A and B, and verbal fluency) as the dependent variables. Sensitivity and specificity of mean relative power for each frequency band was established by plotting receiver operating characteristic (ROC) curves.

## Abbreviations

AD, Alzheimer’s disease; ARWMC, Age-related white matter changes; CT, Computed tomography; EEG, Electroencephalography; FFT, Fast Fourier Transformation; MMSE, Mini mental state examination; MRI, Magnetic resonance imaging; NINDS-AIREN, National Institute of Neurological Disorders and Stroke and Association Internationale pour la Recherché et l’Enseignement en Neurosciences; ROC, Receiver operating characteristic; SIVD, Subcortical ischemic vascular dementia; SIVD-WMH, Subcortical ischemic vascular dementia, based on extensive white matter hyperintensities; VaD, Vascular dementia; WMH, White matter hyperintensities.

## Competing interests

The authors did not receive financial support for the study and do not report any actual or potential conflicts of interest. An abstract of this manuscript was presented as a poster at the 29th International Congress of Clinical Neurophysiology (ICCN), Kobe, Japan.

## Authors’ contribution

ES: Selected, processed and analysed the EEG data and drafted the manuscript. WH: Has been involved in the collection of patient data and has critically appraised the manuscript HW: Has been involved in the collection of patient data and has critically appraised the manuscript. PS: Has made contributions to the conception and design of the study and has critically appraised the manuscript. WF: Has been involved in the storage, identification and retrieval of patient data, has made contributions to the conception and design of the study and has critically appraised the manuscript. FB: Has been involved in the collection of the MRI data and has critically appraised the manuscript. TK: Has been involved in the collection of the cognitive data and has critically appraised the manuscript. CS: Has been involved in the conception and design of the study, the EEG analyses and drafting of the manuscript. All authors read and approved the final manuscript.
